# Supplementary material for: From sequence to enzyme mechanism using multi-label machine learning
Source: BMC Bioinformatics. 2014 May 19;15:150. doi: 10.1186/1471-2105-15-150 (PMC4229970; doi:10.1186/1471-2105-15-150)
Supplement: Additional file 2 — Java code of ml2db. Additional file ml2db_code.tar.gz contains the Java source code to run the multi-label machine learning experiments and save the results to database. The code’s Javadoc is included. [file 1471-2105-15-150-S2.zip › additional file 2/ml2db/utils/doc/uk/ac/ed/inf/utils/database/class-use/DbManagerSingleton.html]

Uses of Class uk.ac.ed.inf.utils.database.DbManagerSingleton


---


|  |  |  |
| --- | --- | --- |
| **Overview** \| **Package** \| **Class** \| **Use** \| **Tree** \| **Deprecated** \| **Index** \| **Help** | |  |
| PREV   NEXT | **FRAMES**    **NO FRAMES**     **All Classes** |  |


---


## **Uses of Class uk.ac.ed.inf.utils.database.DbManagerSingleton**

| Packages that use DbManagerSingleton | |
| --- | --- |
| **uk.ac.ed.inf.utils.database** |  |

Uses of DbManagerSingleton in uk.ac.ed.inf.utils.database

| Methods in uk.ac.ed.inf.utils.database that return DbManagerSingleton | |
| --- | --- |
| `static DbManagerSingleton` | `DbManagerSingleton.getInstance(java.lang.String dbConnPath)` |

---


|  |  |  |
| --- | --- | --- |
| **Overview** \| **Package** \| **Class** \| **Use** \| **Tree** \| **Deprecated** \| **Index** \| **Help** | |  |
| PREV   NEXT | **FRAMES**    **NO FRAMES**     **All Classes** |  |


---
